# Do Extraordinary Claims Require Extraordinary Evidence?

**DOI:** 10.1007/s11406-016-9779-7

**Published:** 2016-10-20

**Authors:** David Deming

**Affiliations:** College of Arts & Sciences, University of Oklahoma, Norman, OK 73019 USA

**Keywords:** Sagan, Anomaly, Paranormal, Hume, Miracle

## Abstract

In 1979 astronomer Carl Sagan popularized the aphorism “extraordinary claims require extraordinary evidence” (ECREE). But Sagan never defined the term “extraordinary.” Ambiguity in what constitutes “extraordinary” has led to misuse of the aphorism. ECREE is commonly invoked to discredit research dealing with scientific anomalies, and has even been rhetorically employed in attempts to raise doubts concerning mainstream scientific hypotheses that have substantive empirical support. The origin of ECREE lies in eighteenth-century Enlightenment criticisms of miracles. The most important of these was Hume’s essay *On Miracles*. Hume precisely defined an extraordinary claim as one that is directly contradicted by a massive amount of existing evidence. For a claim to qualify as extraordinary there must exist overwhelming empirical data of the exact antithesis. Extraordinary evidence is not a separate category or type of evidence--it is an extraordinarily large number of observations. Claims that are merely novel or those which violate human consensus are not properly characterized as extraordinary. Science does not contemplate two types of evidence. The misuse of ECREE to suppress innovation and maintain orthodoxy should be avoided as it must inevitably retard the scientific goal of establishing reliable knowledge.

## Introduction

Over the past few decades the aphorism “extraordinary claims require extraordinary evidence” (ECREE) has been popularized. It has been called “a fundamental principle of scientific skepticism” (Voss et al. 2014: 893) and “an axiom of the skeptical movement” (Goertzel and Goertzel 2015: 292). ECREE is frequently invoked to discredit research dealing with scientific anomalies or any claim that falls outside the mainstream. The statement is usually made without justification or explanation, as if the mere invocation were enough to stifle debate and disqualify any legitimate opposition. Pulverer (2015: 2723) explained, “it is right to expect that particular papers that promulgate extraordinary claims must also be based on the highest levels of evidence and must be subjected to and rise to an extraordinary level of validation.” ECREE has become so ubiquitous that there are instances in the scientific literature where it has been used as the title of a published article without any apparent relation to the content of that article (Light and Warburton 2005; DeVorkin 2010; Hauser and Johnston 2011).

Although the modern roots of ECREE lie in the context of discussing paranormal phenomena (Sagan 1979), it has also been used to discredit mainstream scientific hypotheses. In 2007, a group of geologists hypothesized that a large-scale comet or asteroid impact event in North America 12.9 ka caused the Younger Dryas cooling event. The evidence for this event is significant. Sedimentary layers dating to 12.9 ka contain glass and carbon spherules, are enriched in iridium, and have carbonized material consistent with widespread wildfires (Dalton 2007). Despite the existence of significant corroborating evidence, Pinter and Ishman (2008) characterized the impact hypothesis as an “extraordinary claim” that required “extraordinary evidence.” They concluded that serious consideration of “spectacular stories” would “consume the finite commodity of scientific credibility” (Pinter and Ishman 2008: 38). Yet the consideration of alternative theories is not only allowed in science, but integral to the scientific process itself (Chamberlin 1890).

In other instances, the invocation of ECREE has been virtually unintelligible. Tressoldi (2011: 1) described ECREE as a statement that “is at the heart of the scientific method, and a model for critical thinking, rational thought and skepticism everywhere.” Yet in the same paragraph the author conceded that it was impossible to objectively define the term “extraordinary.” He admitted that “measures of ‘extraordinary evidence’ are completely reliant on subjective evaluation” (Tressoldi 2011: 1). It is clearly impossible to base all rational thought and scientific methodology on an aphorism whose meaning is entirely subjective.

Invocation of the ECREE aphorism tends to confuse more than clarify. Pertinent questions remain unanswered. What is the nature of an extraordinary claim? What qualifies as extraordinary evidence? Should there be two standards of evidence in science? Is there any context in which ECREE can be invoked correctly? In the discussion that follows I argue that the true meaning and proper invocation of ECREE can be understood if its historical roots are traced.

## Carl Sagan and the Paranormal

The current popularity of ECREE originates with its appearance in the book *Broca’s Brain* (1979: 62) by deceased astronomer Carl Sagan. Sagan’s original invocation of ECREE was done largely in the context of discussing the validity of paranormal phenomena such as levitation, visits to Earth by alien spacecraft, astral projection, and the claim that razor blades stored in pyramids retain their edge longer. Unfortunately, Sagan did not define explicitly what constitutes either an extraordinary claim or extraordinary evidence.

The invocation of the term paranormal itself raises a quandary, as the difference between normal and paranormal phenomena necessarily contains a degree of ambiguity. The *Oxford English Dictionary* defines paranormal as “designating supposed psychical events and phenomena such as clairvoyance or telekinesis whose operation is outside the scope of the known laws of nature or of normal scientific understanding.”

There is a profound difference between “events and phenomena” that lay “outside the scope” of the laws of nature and those that are merely beyond “normal scientific understanding.” Everything in nature was originally “beyond normal scientific understanding.” Furthermore, focusing on those aspects of phenomena that are not completely understood is the key to scientific progress. In Thomas Kuhn’s words, “discovery commences with the awareness of anomaly” (1996: 52). The history of astronomy offers one example. The retrograde motion of Mars and changes in its apparent diameter were, at one time, challenges to the Ptolemaic System. A consideration of these anomalies was one factor that led to the eventual adoption of the heliocentric model.

A claim of levitation or telekinesis apparently violates the established laws of nature. But even this category contains ambiguity. The laws of nature are nothing more than inductive generalizations based on the accumulated body of evidence available to science. However we never have all the data. As our observations increase in accuracy or number we may discover that nature operates differently from the manner we formerly supposed.

For more than two centuries Newtonian mechanics was regarded as an accurate description of nature. But in the twentieth century it was discovered that classical mechanics breaks down at relativistic speeds. Unless he was otherwise informed, an individual who had spent his entire life living in a tropical climate would regard a claim that water could turn into a solid as an extraordinary claim that violated the laws of nature as known to him. While it is relatively easy to assess the extent of our knowledge, it is difficult to fathom the depths of our ignorance.

It is doubtful if Sagan would have approved of the use of ECREE to discredit research into anomalous phenomena. He was open to the scientific investigation of both anomalous and paranormal phenomena. Carl Sagan characterized Ian Stephenson’s research on reincarnation as deserving of “further inquiry,” and therefore must have considered the concept plausible (1979: 48). Sagan (1979: 59) considered “scientific aloofness and opposition to novelty” to be “as much a problem as public gullibility.” He organized conferences of the American Association for the Advancement of Science devoted to UFOs and evaluation of Immanuel Velikovsky’s theories. Sagan (1979: 62) believed that “the extraordinary should certainly be pursued,” with “each issue” being “judged on its own merits.” But he also insisted on rigor in scientific method, concluding that “the burden of proof should fall squarely on those who make…proposals” (Sagan 1979: 62).

## The Nature of Proof

A more extreme statement of ECREE was proposed by Marcello Truzzi. In a letter published in the journal *Parapsychology Review*, Truzzi advanced the proposition that “an extraordinary claim requires extraordinary proof” (1975: 24). He later expounded upon the implications of this statement in an article published in *Zetetic Scholar* (1978).

Truzzi’s definition of extraordinary is of little assistance. He stated “something is extraordinary when it is unexpected” (Truzzi 1978: 14). But “unexpected” is not an objective quantity that can be measured. It is a subjective psychological reaction experienced by an observer. Truzzi conceded this when he concluded “the degree to which each of us may be surprised by a strange event is rather relative to our own experience and background” (1978: 15). By defining “extraordinary” in terms of “expectation” Truzzi simply substituted one ambiguous term for another.

Truzzi’s demand for “proof” is also problematical. The word “proof” is usually not used in the context of science. Science is concerned with corroboration and falsification (Popper 1959). But the matter is not that clear. There is no standard handbook of scientific procedure or clear methodological rules. Every scientist in effect makes up his own rules. Acceptance or rejection of scientific knowledge ultimately depends not just on repeatability or corroboration but a human process of social acceptance.

The meaning of the word “proof” depends on context. There are legal proofs, mathematical proofs, and logical proofs. The gist of these is that the concept of proof in various contexts is concerned with “evidence or argument establishing a fact or the truth of anything” (Oxford English Dictionary 2016). A science is a method designed to produce reliable knowledge. Scientists seek truth. But truth itself is difficult to define. The concept of truth has been actively discussed and debated by epistemologists for more than 2,000 years. As the Pyrrhonian skeptics argued in the third century BC, every criterion of truth must itself be validated by a criterion of truth, ad infinitum (Diogenes Laërtius 1905: 415). Every attempt to obtain an objective definition necessarily opens additional doors and reveals new difficulties.

The concept of proof in a legal context may require no more than a probability, a preponderance of the evidence. But when the word proof is invoked in a philosophical sense, it usually connotes absolute certainty. A “proof” in natural philosophy is what the ancient Greeks would have called a “demonstration,” a deductive conclusion derived by analogy from the technique employed in geometry (Deming 2010: 17). The word “proof” is usually not employed in a scientific context because there can be no certainty in an empirical system of knowledge. This was established by presocratic Greek philosophers as early as the fifth or sixth century BC.

One reason that certainty is a logical impossibility in an empirical system is that we can never be sure we have all the data. Another difficulty recognized by the ancient Greeks is that perception is subjective: everyone observes things differently. Heraclitus (c. 540–480 BC) originated the doctrine that there could be no reliable knowledge of sensible things because the natural world was in a state of perpetual change (Deming 2010: 23–24). “Those who step into the same river have different waters flowing ever upon them” (Freeman 1966: 25). Describing the Heraclitean doctrine, Aristotle noted that the followers of Heraclitus “describe all sensible things as ever passing away,” thus there can “be no knowledge of things which were in a state of flux” (Aristotle 1941a: 894). Aristotle concluded that “scientific knowledge is not possible through the act of perception” (1941b: 154). Lacking the technology to make empiricism a practical means of building reliable knowledge, Greek philosophers relied upon deductive logic and the method of logical demonstration derived from geometry. They sought certainty, not probable truth.

Following the invention of the printing press c. 1450, it became possible to improve the reliability of empirical knowledge through continual revision. With the advent of typography, empirical claims and anecdotal data no longer had to either be accepted at face value or immediately rejected. They could be exchanged, sifted, debated, criticized, refined, corroborated, or falsified. “Steady advance implies the exact determination of every previous step; this now became incomparably easier” (Sarton 1962: 66). Thus the Scientific Revolution of the sixteenth and seventeenth centuries was made possible by technological innovation (Deming 2012). The printing press made it feasible to adopt a criterion of repeatability. It became intellectually respectable to accept a pragmatic system of knowledge that established mere probabilities instead of certainties.

In the logical sense, there can be no “proof” in the sciences, extraordinary or otherwise. Truzzi’s demand for “extraordinary proof” is impossible to fulfill for any claim. The statement thus is unintelligible. If ECREE is to have any intelligible meaning, it is best considered in the wording chosen by Carl Sagan.

## Drawing Balls from Urns

One serious philosophical predecessor to Sagan was Pierre-Simon Laplace (1749–1827). In *A Philosophical Essay on Probabilities*, first published in French in 1812, Laplace noted that “the more extraordinary the event, the greater the need of its being supported by strong proofs” (1902: 17).

Laplace was more careful than Sagan, in that he foresaw the necessity of defining what makes an event “extraordinary.” Laplace defined “extraordinary” events in a probabilistic sense as “those classes which include a very small number” (Laplace 1902: 17). He offered the example of an urn containing a million balls, all of which were white in color except one which was black. If a random drawing from the urn produced the black ball, this would qualify as an improbable and thus “extraordinary” event.

Drawing balls from urns is familiar to every student of probability theory. Mathematicians use these examples because the calculation of probabilities is straightforward and exact. But this is never the case in the empirical sciences. Our data are always incomplete and imprecise. We don’t know the nature of the balls in the urn, or how the process of extracting one may be influenced by factors beyond our ken. Characterizing an observation or claim as “extraordinary” without supporting data merely presumes what must be demonstrated. Thus Laplace does little to help us understand the proper context of ECREE in the sciences.

## Ordinary and Extraordinary Evidence

Repeatability is essential to science. It is the pragmatic means by which a system of knowledge based on observations establishes provisional truths. Conceding that “the senses deceive,” Francis Bacon (1858b: 26) advocated reproducibility as a means of overcoming the epistemological limitations of an experimental and inductive philosophy. “Whenever I come to a new experiment of any subtlety (though it be in my own opinion certain and approved), I nevertheless subjoin a clear account of the manner in which I made it; that men knowing exactly how each point was made out, may see whether there be any error connected with it, and may arouse themselves to devise proofs more trustworthy and exquisite, if such can be found” (Bacon 1858b: 30). Science as we know it today was largely defined by the activities of the Royal Society during the seventeenth century. And the members of the Royal Society were aware that any experimental result would have to corroborated. In *History of the Royal Society* (1667: 99), Thomas Sprat noted that the results of experimental trials were subjected to “critical and reiterated scrutiny” until “the whole company has fully satisfied of the certainty and constancy.”

Goertzel & Goertzel have pointed out that while claims deemed “extraordinary” may receive the heightened scrutiny of professed skeptics, “science has a troublesome issue in not being demanding enough of ordinary claims” (2015: 295). There is a growing awareness of a “reproducibility crisis” in science. A survey conducted by the journal *Nature* found that 52 % of researchers agreed that there is “a significant crisis of reproducibility” (Baker 2016: 452). Repeatability in particular is a critical issue in the science of psychology. The Open Science Collaboration (2015: 943) found that only 47 % “of original effect sizes were in the 95 % confidence interval.” The poor reproducibility rate in the psychological sciences was attributed in part to a prioritization of “novelty over replication” (Open Science Collaboration 2015: 943). Innovation is highly valued because it is “the engine of discovery” (Open Science Collaboration 2015: 943). Yet ECREE is often invoked to suppress innovation and support mainstream concepts that themselves may be irreproducible.

## Discrediting Miracles

The origin of ECREE lies in seventeenth and eighteenth-century debates concerning the validity of miracles. Prior to the modern age, people living in Western Civilization were profoundly religious and superstitious. The supernatural world was considered to be real, demonstrable and ordered. Conversely, the phenomenological world revealed by the senses was regarded as transitory, illusory and unworthy of serious study.

In a discussion of the *Etymologies* of Isidore of Seville (560–636), Ernest Brehaut (1873–1953) explained that the intellectual viewpoint of the early Middle Ages in Europe was a mirror image of the modern worldview.

The view held in the dark ages of the natural and supernatural and of their relative proportions in the outlook on life, was precisely the reverse of that held by intelligent men in modern times. For us the material universe has taken on the aspect of order; within its limits phenomena seem to follow definite modes of behavior, upon the evidence of which a body of scientific knowledge has been built up…the attitude of Isidore and his time is exactly opposite to ours. To him the supernatural world was the demonstrable and ordered one. Its phenomena, or what were supposed to be such, were accepted as valid, while no importance was attached to evidence offered by the senses as to the material....it is evident, therefore, that if we compare the dogmatic world-view of the medieval thinker with the more tentative one of the modern scientist, allowance must be made for the fact that they take hold of the universe at opposite ends. Their plans are so fundamentally different that it is hard to express the meaning of one in terms of the other (Brehaut 1912: 51).

For more than a thousand years in Christian Europe the reality of miracles was unquestioned. The miracles of Jesus Christ were taken as substantive proof of his divinity. Among other feats recorded in the Gospels, Jesus turned water into wine (*John* 2.1–2.11), walked on water (*Mark* 6.45–6.52), and raised the dead from the tomb (*John* 11.1–11.44).

The most influential of the Fathers of the Western Christian Church was Augustine of Hippo (354–430). In *City of God*, Augustine affirmed that miracles were not limited to the time of Jesus but were commonplace in his own time: “even now miracles are wrought in the name of Christ” (Augustine 1899: 485). Prodigies recorded by Augustine included miraculous cures of blindness, breast cancer, gout, paralysis, and demonic possession (1899: 485–487). Augustine even listed multiple instances of the dead being restored to life (1899: 488–489).

Before the Christian age, the Greeks and Roman were also remarkably superstitious. George Sarton characterized their “firm belief in divination” as “the outstanding superstition of classical antiquity (1960: 464). The histories of Alexander the Great (356–323 BC) by Arrian (c. 86–186 AD) and Diodorus Siculus (fl. 1st century BC) are replete with repeated instances of important people drawing serious inferences from superstitious omens.

Following a victory at the Battle of the Granicus River in 334 BC, Alexander consolidated his position by marching down the coast of Ionia and conquering city-states under the control of the Persian empire. At Miletus, Alexander was unsure if he should attack by sea or land. The critical tactical decision was based upon the subjective interpretation of a superstitious omen. “An eagle had been seen sitting upon the shore, opposite the sterns of Alexander’s ships…[Alexander] admitted that the eagle was in his favor; but as it was seen sitting on the land, it seemed to him rather to be a sign that he should get the mastery over the Persian fleet by defeating their army on land” (Arrian 1893: 47–48).

Making important decisions on the basis of superstition could have devastating consequences, even to the point of crippling an entire polity. On the night of August 27, 413 BC, an eclipse of the Moon prevented the Athenian navy from fleeing Syracuse. Subsequently, the Athenians suffered a complete defeat at the hands of the Syracusans, and Athenian power was broken forever (Grote 1899: 147–151).

Roman culture was similarly preoccupied with superstitious beliefs. In *Decline and Fall of the Roman Empire*, Edward Gibbon described the Romans as being possessed by “a puerile superstition that disgraces their understanding” (1909: 318).

They listen with confidence to the predictions of haruspices, who pretend to read in the entrails of victims the signs of future greatness and prosperity; and there are many who do not presume either to bathe, or to dine, or to appear in public, till they have diligently consulted, according to the rules of astrology, the situation of Mercury and the aspect of the Moon (Gibbon 1909: 318).

With the rise of empiricism during the Renaissance, superstitious beliefs began to wane. The European embracement of empiricism was quite contrary to the viewpoint common amongst the Greek philosophers. In *Theaetetus*, Plato quoted Socrates as asserting “no one knows whether what appears to him is the same as what appears to another, and everyone knows that what appears to himself in one way at one time appears to him differently at another” (Burnet 1920: 239). In Plato’s view, nothing related to the senses or dealing with observation could be an object of scientific knowledge. “Whether a man gapes at the heavens or blinks on the ground, seeking to learn some particular of sense, I would deny that he can learn, for nothing of that sort is [a] matter of science” (Plato 1937: 789).

The experimental method was known to the ancient Greeks, but their experiments tended to be limited and anecdotal rather than systematic. The subjugation of reason to observation began in Europe during the thirteenth century. Roger Bacon argued that “reasoning does not suffice, but experience does” (Bacon 1928: 583). His *Opus Majus* contained an entire section devoted to experimental science. One reason that Europeans turned to empiricism was their contemplation of the properties of the magnet. The existence of lodestones suggested that nature contained occult forces and properties that could never be apprehended by logical reasoning alone. Bacon concluded that rational proofs alone were insufficient because “all things must be verified by experience” (1928: 584).

In an age in which every serious European scholar was also a theologian, any activity that from a presentist perspective would qualify as scientific had to compatible with Christian orthodoxy. Observation of the natural world was not only allowable, it was a virtual requisite for natural theology. The door had been opened by Paul the Apostle (c. 0–60 AD). In *Romans* (1.20), Paul wrote that God could be known through the study of nature. “For the invisible things of him from the creation of the world are clearly seen, being understood by the things that are made, even his eternal power and Godhead; so that they are without excuse.” After the Bishop of Paris condemned metaphysical speculation in 1277, scholars and theologians turned to empiricism partly out of necessity (Deming 2010: 156).

In the seventeenth century, experimental philosophy bloomed under the auspices of the Royal Society in England (Deming 2012: 205–211). Aristotelean natural philosophy withered. In *Academiarum Examen* (1654: 67) John Webster condemned Aristotelean philosophy as “merely verbal, speculative, abstractive, formal and notional, fit to fill the brains with monstrous and airy chimeras, speculative, and fruitless conceits.”

As empirical evidence became the accepted standard of proof, people began to question the validity of miracles. Among the first to openly question the reality of the miraculous was the Dutch philosopher, Baruch Spinoza (1632–1677). In *Tractatus Theologico-Politicus* (1670), Spinoza asserted that natural law had been established by God and was therefore immutable. “Nature cannot be contravened…she preserves a fixed and immutable order” (Spinoza 1887: 82). Spinoza attributed miracles to human ignorance. “A miracle is an event of which the causes cannot be explained by the natural reason through a reference to ascertained workings of nature” (Spinoza 1887: 84). In fact, a claim that the laws of nature had been overcome was tantamount to an assertion “that God acted against His own nature--an evident absurdity” (Spinoza 1887: 83).

Among those influenced by Spinoza was the Huguenot skeptic Pierre Bayle (1647–1706). In *Miscellaneous Thoughts on the Comet of 1680*, Bayle (1708: 450) discounted accounts of the miraculous. “We must never have recourse to miracle, when we may explain by natural reasons, “ because ”our schools of theology, as well as those of philosophy, teach us not to multiply beings or miracles without a necessity.” In his enormously influential *Historical and Critical Dictionary*, first published in 1697, Bayle suggested that miracles were not genuine instances of the suspension of the laws of nature, but rooted in human credulity and gullibility (1710: 1766).

Protestants embraced empiricism when it helped them discredit Catholicism. In *A Discourse Against Transubstantiation* (1684), John Tillotson argued for the validity of sense perception. “If we be not certain of what we see, we can be certain of nothing” (Tillotson 1684: 3). Tillotson concluded that the supposed miracle of transubstantiation was “a most self-evident falsehood” (1684: 2). Others sought consilience between science and religion. In *A Discourse of Miracles*, John Locke (1632–1704) acknowledged that a miracle was necessarily defined to be an operation “contrary to the fixed and established laws of nature” (1824: 264). But then Locke warned that the laws of nature were not completely known. Before a man could judge that an event was truly a miracle “he must know that no created being has a power to perform it” (Locke 1824: 264). Therefore it was possible, in effect, to preserve the validity of religion through miraculous testament without violating natural law.

As the Enlightenment of the eighteenth century proceeded, revelation and the miraculous came under attack and apologetics were proffered in their defense. Writing in 1740, the Anglican latitudinarian Arthur Ashley Sykes conceded that miraculous events required substantiation by “extraordinary proof.” “Where there is only an account of extraordinary facts related, without any extraordinary proof of their being true, the credibility of them is lessened even by the extraordiness of the facts” (Sykes 1740: 206).

But Sykes was unwilling to conclude that the miracles recorded in the Bible were fictions. He argued that the credibility of Christian miracles originated in the genuine inspiration of the writers who recorded them. The best proof of this was the fulfillment of Biblical prophecy. “Prophecies…in Scripture do contain the foretelling of many future events: the accomplishment of these events is the evidence to us of the truth of the revelation itself” (Sykes 1740: 208).

Sykes was not alone in his regard for the importance of Biblical prophecy. Isaac Newton believed that the fulfillment of Biblical prophecy was evidence for God’s providential rule of the world. Much of Newton’s time in theological research was spent in trying to decipher prophecies in the Books of *Daniel* and *Revelation*. His interpretation of these texts was published posthumously in 1733 as *Observations Upon the Prophecies of Daniel, and the Apocalypse of St. John*.

As the Age of Reason advanced, the apologetics became more strained. In 1749, English clergyman Conyers Middleton conceded that “ordinary facts, related by a credible person, furnish no cause of doubting from the nature of the thing: but if they be strange and extraordinary; doubts naturally arise, and in proportion as they approach towards the marvelous” (1749: 217).

Middleton’s argument for preserving belief in miracles was that the age of the miraculous had been closed. The miracles performed by Christ and his Apostles were real, but there had been no genuine miracles since this time. “There is no sufficient reason to believe, from the testimony of antiquity, that any miraculous powers did ever actually subsist in any age of the Church, after the times of the Apostles” (Middleton 1749: xci).

Middleton’s argument is apparently a special pleading. But closing the age of the miraculous is consistent with closing the age of revelation. In Judaism, Christianity, and Islam, the age of prophecy is considered to be over, and the revelations of the prophets are regarded as final and complete. It was thus logically consistent to argue that the age of miracles had also been concluded.

Among those who attacked the credibility of the miraculous was the editor of the French *Encyclopédie*, Denis Diderot. Diderot embraced skepticism, questioned the authenticity of Christianity, and leaned toward atheism. He dismissed the reality of miracles, concluding “all those who saw miracles there had made up their mind to see them” (Diderot 1916: 61).

The most significant of the Enlightenment attacks on the reality of miracles was the essay *On Miracles* (1748) by the Scottish writer David Hume. It is in Hume’s essay that we find a definitive characterization of ECREE as a balancing of the evidence. If “the fact…partakes of the extraordinary and the marvelous…the evidence…received a diminution, greater or less, in proportion as the fact is more or less unusual” (Hume 1748: 179).

Hume explained that there must be “a contest of two opposite experiences” (1748: 179). Miracles required extraordinary proof or evidence because, by definition, a miracle was “a violation of the laws of nature; and as a firm and inalterable experience has established these laws, the proof against a miracle, from the very nature of the fact, is as entire as any argument from experience can possibly be imagined” (Hume 1748: 180).

As an example of a miracle, Hume offered the claim that a piece of lead would remain suspended in air when released. Because human beings had observed the contrary innumerable times over the ages, in order for the claim to be believed, the number of observations substantiating the supposed miracle must be greater. If people had observed a million times that a piece of lead will fall to the ground when released, then to establish the claim that a piece of lead will remain suspended in air requires a million and one observations. A hundred-thousand testaments of the defiance of gravity will be insufficient, because these must be weighed against a million contradictions.

Thus Hume defined precisely what is meant by “extraordinary” evidence or proof. “Extraordinary” means numerous. “Extraordinary” evidence is not a separate category or type of evidence, it is an extraordinarily large number of observations. “Extraordinary” evidence is only required when it must be balanced against a very large number of contrary observations.

The crux of the matter is that in order to properly characterize a claim as “extraordinary,” there must exist weighty evidence of the *exact* antithesis. A claim or theory is not “extraordinary” solely because it is novel, unusual, or is in disagreement with human consensus. The claim that a rock will remain suspended in air when released from the hand is extraordinary because we have an extraordinary number of observations to the contrary. But a claim that it is possible to construct and operate a heavier-than-air flying machine is not “extraordinary,” even though we have overwhelming evidence that objects heavier than air fall to the ground. The two instances are not exactly comparable. A heavier-than-air flying machine is an object, but it is a unique object. Objects that we may have observed falling to the ground, such as stones, do not have engines or wings. It does not matter that we may have observed falling stones ten million times: a stone is not an airplane.

Similarly, a claim to achieve heat generation through cold fusion is not “extraordinary,” simply because no one has done it before. The claim can only be “extraordinary” if there have been a very large number of previous trials in which the experiment has failed. And the experimental apparatus and circumstances in these previous trials must have been not merely similar, but identical in all respects. If even one parameter has changed, the balancing of the evidence is no longer a thousand-to-one against heat generation, but one-to-zero in favor of heat generation.

## Conclusion

The confusion regarding what is meant by an “extraordinary claim” arises in simple carelessness. Carl Sagan did not define the term “extraordinary.” This allowed others to arbitrarily characterize as “extraordinary” any idea or claim that violated majority opinion. This unfortunate debacle could have been avoided if Sagan had simply been more precise. As long ago as the fifth century BC, Socrates established the rule that intelligible discussions of serious matters require exact definitions. Confusion arising from ambiguities in language is one of Francis Bacon’s Four Idols, biases that hinder objective science. Bacon explained “the ill and unfit choice of words wonderfully obstructs the understanding…words plainly force and overrule the understanding, and throw all into confusion, and lead men away into numberless empty controversies and idle fancies” (1858a: 55).

The proper origin and explanation of ECREE is found in David Hume’s essay *On Miracles*. It is in this work that the exact definition of the term “extraordinary” is found. An “extraordinary” claim is one that is contradicted by a massive amount of existing evidence. However there must be an exact correspondence between experiences before a claim can legitimately be characterized as “extraordinary.” Ideas, theories, or observations that are merely novel are not “extraordinary,” nor do they require an “extraordinary” amount of evidence for corroboration. Science does not contemplate two types of evidence. The misuse of ECREE to suppress innovation and maintain orthodoxy should be avoided as it must inevitably retard the progress of science in establishing comprehensive and systematic bodies of reliable knowledge.
